# Pupillary Hippus as a Biomarker: Spectral Signatures and Complexity Approaches in Autonomic and Clinical Contexts

**DOI:** 10.3390/bioengineering12121376

**Published:** 2025-12-18

**Authors:** Vincenzo Rizzuto, Marco Laurino, Roberto Montanari, Angelo Gemignani, Michele Figus, Giuseppe Covello, Niccolò Candelise, Davide Borroni, Guna Laganovska, Luca Mesin

**Affiliations:** 1 International School of Advanced Studies, University of Camerino, 62032 Camerino, Italy; vincenzo.rizzuto@studio.unibo.it; 2 Department of Surgical, Medical and Molecular Pathology and Critical Care Medicine, University of Pisa, 56126 Pisa, Italy; angelo.gemignani@unipi.it (A.G.); michele.figus@unipi.it (M.F.); giucovello@gmail.com (G.C.); 3 Department of Ophthalmology, Riga Stradins University, LV-1007 Riga, Latvia; borroni@eyemetagenomics.com (D.B.); glaganovska@ml.lv (G.L.); 4 Institute of Clinical Physiology, National Research Council, 56124 Pisa, Italy; marco.laurino@cnr.it; 5 Institute of Pharmacology, Heidelberg University Hospital, 69120 Heidelberg, Germany; roberto.montanari@pharma.uni-heidelberg.de; 6 Molecular Medicine Partnership Unit, European Molecular Biology Laboratory (EMBL), 69120 Heidelberg, Germany; 7 Department of Neuroscience, University of Pisa Hospital, 56126 Pisa, Italy; 8 Ophthalmology, Department of Medical and Surgical Specialties, Azienda Ospedaliero-Universitaria Pisana, 56124 Pisa, Italy; 9 Independent Researcher, 00162 Rome, Italy; niccolo.candelise@gmail.com; 10 Clinic of Ophthalmology, P. Stradins Clinical University Hospital, LV-1002 Riga, Latvia; 11 Mathematical Biology and Physiology, Department of Electronics and Telecommunications, Politecnico di Torino, 10129 Turin, Italy

**Keywords:** pupillary hippus, pupillary unrest, spectral analysis, frequency bands, complexity, entropy, pupillometry, autonomic biomarkers, clinical applications, glaucoma

## Abstract

Pupillary hippus, i.e., the spontaneous bilateral oscillation of pupil diameter under constant illumination, provides a non-invasive window into autonomic and central nervous system dynamics. Despite decades of research, a unified framework linking its spectral features, physiological underpinnings, and analytical complexity is still missing. In this narrative review, we synthesize spectral and nonlinear pupillometry studies to propose a paradigm-aware taxonomy of hippus frequency bands that integrates evidence from autonomic tests and cognitive-arousal paradigms. Across the literature, low-frequency components tend to covary with respiratory/vasomotor autonomic rhythms, while higher-frequency fluctuations and complexity indices are more sensitive to cognitive load, visual fatigue, and pathological states but remain methodologically heterogeneous. Finally, we explore contexts in which hippus-based biomarkers show clinical potential, with a main focus on glaucoma as an emerging translational model, while underscoring the methodological and translational gaps that currently hinder their validation and routine clinical adoption. Physiology, spectral metrics, and complexity-based analyses are integrated to lay the foundations of a coherent framework.

## 1. Introduction

Pupillary hippus, also referred to as pupillary unrest, denotes spontaneous bilateral oscillations of pupil diameter, typically ranging from 0.04 to 2 Hz, with a modal frequency near ∼0.3–0.5 Hz, although precise boundaries vary with experimental conditions and analysis methods. These oscillations persist under steady illumination and fixation, reflecting the dynamic interplay of autonomic and central control mechanisms [1,2,3,4]. While the pupillary light reflex has long been used to assess afferent and efferent integrity [5,6], modern pupillometry extends far beyond a simple photic response, encompassing influences from the autonomic, cognitive, and affective domains [7,8,9,10,11,12,13]. Consequently, hippus represents a multideterministic signal, emerging from both brainstem reflexes and higher-order regulatory circuits, whose analysis requires approaches that capture both its spectral and nonlinear properties [14,15,16].

The physiological control of pupil diameter involves a dual innervation system. The Parasympathetic Nervous System (PNS) pathway, originating in the Edinger–Westphal nucleus, drives constriction of the sphincter muscle, whereas the Sympathetic Nervous System (SNS) pathway, projecting from the hypothalamus and locus coeruleus (LC) to the superior cervical ganglion, governs dilation of the radial dilator muscle [4,17]. These two systems, although anatomically distinct, are continuously co-regulated by supranuclear centers. The LC plays a particularly critical role: it simultaneously activates the sympathetic dilator pathway and inhibits parasympathetic constriction at the Edinger–Westphal nucleus, integrating arousal, vigilance, and cognitive load into moment-to-moment pupil dynamics [5,18,19]. Under steady luminance, cortical imaging indicates that pupil size tracks cholinergic dynamics, whereas the temporal derivative of the pupil correlates more tightly with noradrenergic dynamics, refining the interpretability of spontaneous fluctuations [16]. Dysfunctions in this autonomic balance have been documented in various clinical populations, including temporomandibular disorder patients, where pupillometric evidence revealed altered sympathetic–parasympathetic modulation and descending pain system dysregulation [20,21]. Thus, the pupil could provide an entry point to brainstem and cortical activity [7,11].

Hippus is believed to arise from the ongoing interaction between these sympathetic and parasympathetic loops. It displays nonlinear temporal characteristics and broad-band oscillations whose frequency and amplitude vary with arousal state, respiration, cardiac cycles, and resting-state neural activity [2,3,14,22,23,24]. This coupling suggests that pupil fluctuations share central regulatory mechanisms with other autonomic rhythms. Reported frequency boundaries vary widely across studies and methods. Similarly, the absence of standardized metrics has hindered comparisons between research groups and slowed clinical translation [19,25]. Analogies with HRV-like LF/HF banding (i.e., low/high frequency ratio of heart rate variability) are heuristic rather than established in pupillometry; physiological attributions should be validated with targeted paradigms and pharmacology [2,19,25].

In this context, spectral and complexity-based analyses offer complementary perspectives. Spectral decomposition may reveal frequency-specific contributions of sympathetic and parasympathetic inputs, whereas complexity measures, such as approximate or sample entropy, can quantify the degree of irregularity and adaptability of the underlying control system [3,26]. Integrating these approaches may allow a more comprehensive description of the signal, bridging physiology, cognitive neuroscience, and clinical applications.

This review therefore aims to provide a conceptual framework for pupillary hippus as a physiological and potentially clinical biomarker. First, we revisit the physiological organization of pupillary oscillations and their modulation by central autonomic pathways. We then propose a paradigm-aware spectral taxonomy, summarizing evidence from autonomic perturbation, cognitive, and visual-fatigue paradigms. Finally, we introduce complexity analysis for pupillometry and discuss translational opportunities for clinical application.

**Literature overview approach.**

This narrative synthesis is based on iterative literature searches conducted between January 2020 and October 2025 in PubMed/MEDLINE and Scopus using a core combination of terms for spontaneous pupillary oscillations (pupillary hippus/pupillary unrest/spontaneous pupillary oscillations) and for quantitative analysis (spectral/frequency/ power spectral/complexity/entropy/nonlinear). With this core search string, we retrieved 35 records in PubMed/MEDLINE and 11 records in Scopus.

These records provided an initial nucleus of hippus-focused studies that explicitly addressed spectral or nonlinear properties of pupil dynamics.

From this nucleus, we then adopted a deliberately thematic and iterative strategy. First, we performed backward–forward citation tracking on the included articles to identify pre-2020 seminal work on pupillary physiology, pharmacology, autonomic modulation, and early descriptions of pupillary unrest. Second, we ran targeted domain-specific searches (e.g., combining pupillometry terms with “glaucoma”, “ipRGC”—i.e., Intrinsically Photosensitive Retinal Ganglion Cell—“autonomic”, “LC/NE system”, “sleep”, “anesthesia”, and “mTBI”) to map related studies that are conceptually relevant for interpreting hippus but are not always indexed under hippus-specific keywords. This iterative “branching” process substantially expanded the corpus well beyond the articles captured by the initial core string.

Rather than aiming for a fully systematic review of all pupillometry applications, we applied a paradigm-based screening. Titles and abstracts were evaluated for conceptual relevance to spontaneous low-frequency hippus under steady illumination and to its quantitative analysis.

For Section 4 (Spectral Analysis of Hippus), we included only studies that (i) recorded pupil dynamics under constant luminance and fixation; and (ii) explicitly reported spectral bands or frequency ranges together with a proposed physiological attribution and/or correlations with other autonomic signals (e.g., HRV, respiration, and EDA). After screening, 31 articles met these criteria and were used to build the paradigm-aware spectral taxonomy.

For Section 5 (Nonlinear Analysis of Pupil Dynamics), we focused on key methodological contributions applying nonlinear or complexity measures to pupillometry. We retained 21 articles that either (i) introduced or adapted nonlinear estimators (e.g., ApEn, SampEn, DFA, RQA, and fractal dimension) to pupil data or (ii) provided substantive empirical applications illustrating their psychophysiological or clinical interpretability.

For Section 6 (Clinical and Translational Applications), we adopted a broader lens regarding clinical pupillometry, summarizing established applications mainly based on light-evoked PLR metrics and then narrowing our discussion to glaucoma as an emerging translational model for integrating ipRGC-mediated PLR/PIPR measures with hippus-based spectral/complexity indexes.

## 2. Physiology of Pupillary Oscillations

### 2.1. Ocular Anatomy and Efferent Control

The pupil is a circular aperture at the center of the iris (typical human range ∼2–8 mm); the iris sphincter mediates constriction, and the radial dilator mediates dilation [5,7]. Final efferent control is dual: parasympathetic preganglionic neurons from the Edinger–Westphal nucleus project via the oculomotor nerve to the ciliary ganglion (sphincter), whereas sympathetic premotor pathways descend from hypothalamus and locus coeruleus (LC) to the intermediolateral column and superior cervical ganglion (dilator) [4,17,18]. Although anatomically distinct, these routes are co-modulated by supranuclear circuits involved in arousal and orienting; noradrenergic mechanisms can facilitate sympathetic outflow and suppress parasympathetic drive at or upstream of the Edinger–Westphal nucleus [18].

### 2.2. Central Integration and Orienting Circuitry

Beyond the classical light reflex, midbrain and cortical systems contribute to rapid context-dependent pupil changes. In particular, primate microstimulation studies show that the intermediate layers of the superior colliculus can causally drive the transient pupil dilation that accompanies orienting responses, likely by engaging brainstem circuits that suppress Edinger–Westphal parasympathetic output and/or recruit sympathetic pathways [27]. Causal evidence further indicates that the superior colliculus jointly coordinates saccadic and pupillary orienting responses, with global luminance modulating the pupil component more than the saccadic component, consolidating its role in non-luminance-mediated pupil control [28]. Multi-site recordings in non-human primates additionally show that spiking in locus coeruleus, superior colliculus, and cingulate cortices is time-locked to spontaneous fluctuations in pupil diameter during fixation, indicating that orienting and arousal circuits jointly shape ongoing pupil dynamics [27,29,30]. During sustained cognitive processing, dilation reflects joint parasympathetic withdrawal and sympathetic engagement, not a purely sympathetic phenomenon [31]. More broadly, modern pupillometry shows that pupil behavior integrates illumination, autonomic state, and cognitive–affective drives [6,7,8,32].

### 2.3. Nonlinear Dynamics and Coupling to Autonomic Rhythms

Hippus exhibits a nonlinear temporal structure with broad-band oscillations modulated by arousal; irregular/quasi-chaotic features have been described [14,33]. Slow fluctuations can couple with respiration and cardiovascular rhythms: early and later reports showed respiratory-phase coupling in animals and humans and cross-spectral relations with low-frequency autonomic components [3,22,23,34,35]. Baroreflex involvement has been inferred from these relationships, although paradigms and analyses vary across studies. Guided or meditative breathing is associated with increased low-frequency spontaneous pupillary oscillations [36]; however, the current human evidence that breathing systematically shapes pupil dynamics is inconclusive, and claims of direct entrainment should be made cautiously [34].

### 2.4. LC–Basal Forebrain Neuromodulatory Network

Two neuromodulatory systems are central to arousal-linked pupil fluctuations: the noradrenergic locus coeruleus (LC) and the cholinergic basal forebrain (BF). Under constant luminance, cortical axonal imaging in awake mice shows that rapid pupil dilations closely follow phasic noradrenergic activity, whereas longer-lasting dilations during sustained arousal (e.g., locomotion) are accompanied by tonic cholinergic activity, indicating that pupil dynamics report the combined state of these neuromodulatory systems [16]. Thus, pupil size provides a sensitive—but neither exclusive nor perfectly specific—readout of LC/BF and broader ascending arousal system (AAS) activity, and its interpretation must take into account task context, global arousal state, and the temporal scale of analysis [16,37,38]. In macaques, multi-site recordings show pupil-locked spiking not only in LC but also in superior colliculus and cingulate cortex, supporting a distributed arousal-orienting network rather than a single-nucleus driver [27,29,30]. Moreover, although the pupil–LC relationship is approximately monotonic across large changes in pupil size, it is strongly state-dependent and too variable to serve as an accurate real-time readout of LC spiking, cautioning against fine-grained interpretations of single-trial pupil traces [39].

### 2.5. Pharmacological Dissociation of Autonomic Components

Pharmacological manipulations help to disentangle autonomic contributions to hippus. In healthy participants, a parasympatholytic (e.g., tropicamide) reduces pupillary unrest, whereas a sympathomimetic (e.g., phenylephrine) has little effect on the spontaneous oscillations per se, suggesting that hippus magnitude depends more strongly on parasympathetic input than on absolute sympathetic tone under steady luminance [2]. Complementarily, μ-opioid agonists (e.g., fentanyl/remifentanil) depress pupillary unrest/Pupillary Unrest Index (PUI) in ambient light, consistent with central μ-opioid suppression of locus coeruleus firing and reduced parasympathetic–sympathetic interplay at the Edinger-Westphal level [40]. These findings are congruent with models in which hippus emerges from the ongoing interaction of PNS/SNS loops with central modulatory control.

## 3. Measurement and Reporting Standards

Accurate quantification of pupillary hippus demands rigorous methodological control. Variability in illumination, sampling, preprocessing, and physiological context has historically hindered cross-study comparability [19,25]. To facilitate reproducibility and translation, this section summarizes the minimal reporting requirements and technical standards aligned with recent recommendations [19,25,41].

- Illumination and visual setup
  Report ambient and display luminance (cd· m^−2^), the measurement device/procedure, monitor model/refresh, and whether temporal light modulation (TLM) sources (e.g., PWM in displays/lighting) and auto-exposure were disabled. Include, when available, a temporal spectrum or verification of the light source (e.g., oscilloscope/stroboscopic meter) because TLM can inject spurious power in the hippus range and should be reported along with luminance (cd· m^−2^), instrument, and setup. Maintain constant luminance and use a fixed far-vision target to avoid near-response confounds; report the IR illuminator wavelength (e.g., 850–940 nm). These practices are consistent with pupillography standards and TLM/flicker best-practice guidance [19].
- Fixation, accommodation, and head stabilization
  State target distance/size/contrast and accommodative demand (diopters); moreover, use chin/forehead rest and report monocular vs. binocular capture. Minimize eye movements or define a gaze window. If gaze is recorded, correct for pupil-foreshortening error (PFE) using geometric/ellipse models or include gaze angle as a regressor and provide the gaze-angle distribution [42]. If PFE is corrected, report residual error after correction and describe the correction method and calibration layout [42,43].
- Sampling and exposure
  Report camera model, sampling rate (Hz), exposure, IR wavelength, and firmware. For hippus (with most of the bandwidth ≲ 2 Hz), using a sampling frequency of 30 Hz is an acceptable minimum (far above the Nyquist limit and useful to resolve even small fluctuations and higher-frequency noise and artifacts, e.g., blinks, saccades, and eyelid jitter); use ≥60–120 Hz when computing derivatives, handling fast artifacts (blinks/saccades), or performing tight multimodal synchronization.
  If data are down-sampled, apply and document anti-alias filtering before resampling [44]. Pair acquisition choices with preprocessing guidelines if up-/resampling is applied [25,45]. Justify any sampling frequency <30–60 Hz, noting its impact on spectral fidelity and blink-edge artifacts. Avoid frame averaging unless signal-to-noise justification is reported.
- Calibration and geometry
  Describe pixel-to-millimeter conversion, calibration artifact, eye-camera geometry, and pupil-fitting method (ellipse vs. circle). If reporting data in mm, describe the calibration artifact or geometric model used; otherwise, specify that you work in consistent arbitrary units. If output remains in arbitrary units, explicitly state that identical scaling was maintained across conditions [19]. State use of corneal-reflex tracking, head stabilization, and which eye(s) was (were) recorded [19].
- Vigilance and circadian control
  Record time-of-day, sleep, caffeine/nicotine intake, and medication; include subjective alertness ratings. Hippus and pupillary unrest are vigilance-sensitive, increasing under drowsiness and darkness [46,47]. Unless vigilance is the target variable, recordings should avoid drowsy states and remain shorter than ∼5 min under constant illumination [19,25]. Participant demographics and environment (temperature and background noise) should also be reported [25].
- Physiological covariates (respiration and cardiovascular)
  When examining autonomic coupling, report respiratory (belt/flow) and cardiovascular sensors (e.g., electrocardiogram ECG and photoplethysmography PPG), their sampling rates, and synchronization. Define analytic methods (cross-spectra, coherence, and phase-locking) and describe breathing protocols and any baroreflex-relevant manipulations. If paced breathing or apnea paradigms are used, specify frequency (e.g., in cycles per minute, 6 cpm ≈ 0.1 Hz) and duration, enabling cross-modal comparison with HRV [48,49].
- Preprocessing
  Provide a preprocessing log: blink/saccade detection criteria, removal and maximum gap interpolated (e.g., ≲150 ms), interpolation method, detrending, and filters (type/order/cut-offs). For spontaneous hippus, avoid over-high-pass filtering (>0.03–0.05 Hz), which attenuates LF content. Report % missing and % interpolated per epoch; preprocessing scripts and parameter files should be shared to support replication [45]. Report whether anti-alias filters were applied during down-sampling [25,44]. Account for blink-locked pupil responses, which can mimic low-frequency unrest [50].
- Outcome metrics
  Define spectral bands with physiological rationale (e.g., LF∼0.04–0.25 Hz; HF∼0.25–2 Hz, adaptable by paradigm) and specify the Power Spectral Density (PSD) estimator (Welch/AR/multitaper), window/overlap, leakage control, and normalization (absolute vs. % power). For complexity, state the algorithm and parameters (e.g., for methods searching recurrences, e.g., sample entropy SampEn, approximate entropy ApEn and Recurrence Quantification Analysis RQA, the embedding dimension m, the tolerance r, the duration of the epochs N, the norm measuring distance between points; for methods to explore long-range correlations and scaling like Detrended Fluctuation Analysis DFA, the number of scales and the order of the polynomial for the detrend), stationarity checks, and missing-data policy; align parameter choices with established preprocessing guidance [41]. When inferring autonomic contributions under steady light, contextualize with pharmacology: parasympatholysis (tropicamide) reduces/abolishes hippus; sympathomimetics (phenylephrine) typically dilate without suppressing unrest; μ-opioid agonists depress PUI/hippus [2,40].
- Minimum reporting checklist
  Because methodological heterogeneity has long hindered comparability across pupillometric studies, we outline below a concise reporting checklist summarizing the minimum technical and procedural details required for transparent replication and interpretation of hippus-related findings.
  Transparent methodological reporting will enable quantitative meta-analysis of hippus spectra and promote standardization across pupillometric studies (Table 1).

**Table 1:** Recommended reporting domains for pupillary hippus studies.

Domain	Required Reporting	Notes and Key Sources
Luminance	Ambient + display luminance (cd·m^−2^); measurement device/procedure; monitor model/refresh; TLM/PWM and auto-exposure status	Keep constant; report IR illuminator wavelength (e.g., 850–940 nm) and disable PWM/flicker on displays/lighting [19].
Fixation/Accommodation	Target distance/size/contrast; diopters; head rest; mono vs. binocular	Avoid near-task changes during baseline; keep accommodative demand constant [19,43].
Eye position/PFE	Gaze window; PFE correction/regression if gaze available; gaze-angle distribution	State correction method/fit model; report gaze calibration error (°) [42,43].
Sampling	Camera model; sampling rate (Hz); exposure; IR wavelength; firmware	≥30 Hz minimum for hippus; use ≥60–120 Hz if derivatives, >1 Hz content, fast artifacts, or tight multimodal synchrony; avoid motion blur; state synchronization method [25,44].
Calibration/Geometry	Pixel to mm conversion; eye–camera distance/angle; pupil fit (ellipse vs. circle); corneal reflex; recorded eye(s)	State head stabilization (chin/forehead rest) [19].
Vigilance	Time-of-day; recent sleep/caffeine; alertness scale/test	Hippus/PUI are vigilance-sensitive (dark-room pupillographic sleepiness testing—PST—evidence) [25,46,47].
Resp/HR	Sensors (RESP, ECG/PPG), sampling, synchronization; analysis method	Cross-spectra/coherence/phase-locking; report breathing rate; note baroreflex-relevant manipulations; anchor HRV banding when drawing analogies [48,49].
Preprocessing	Blink/saccade rules; max-gap interpolation; filters (type/order/cut-offs); %missing/%interpolated	Avoid high high-pass (>0.03–0.05 Hz) for LF hippus; anti-alias before down/up-sampling; account for blink-locked pupil response; share code/parameters [25,45,50].
Spectral metrics	LF/HF band edges; PSD estimator; window/overlap; leakage/taper; normalization	Justify band edges physiologically and per paradigm (respiration/vigilance); state Welch/AR/multitaper choices; see Section 4.7 for proposed paradigm-aware taxonomy [2].
Complexity metrics	Algorithm + parameters (ApEn/SampEn: *m*, *r*, *N*; DFA/RQA settings); stationarity; missing-data policy	Effect of epoch duration, sample rate, low-frequency trends [51]; SampEn often ≥ ApEn in practice [26,33].
Pharmacology	Tropicamide/phenylephrine; μ-agonists; (optionally caffeine/nicotine)	Under steady light: PNS block ↓ or abolishes hippus; μ-agonists ↓ PUI/hippus [2,40].
Quality and Sharing	Sex/age, inclusion rules; software/code	Reproducibility [25].

## 4. Spectral Analysis of Hippus

### 4.1. Frequency Ranges and Candidate Mappings

Under steady luminance and fixation, most spontaneous pupillary power (hippus) lies between ≈0.04 and 2 Hz, with a broad mode near ≈0.3 Hz; the exact limits vary with illumination, task, recording duration, and the spectral estimator used [2]. Across paradigms, several banding schemes recur. In PST, slow fluctuations up to ≤0.8 Hz, i.e., the domain of the PUI, are emphasized under prolonged darkness and fixation [19,52]. Autonomic and respiratory studies typically adopt a low-frequency/high-frequency (LF/HF) framework [2,48], whereas some cognitive and affective paradigms extend the high-frequency cut-off to capture luminance-independent components of mental effort and arousal [53,54].

**Figure 1:**
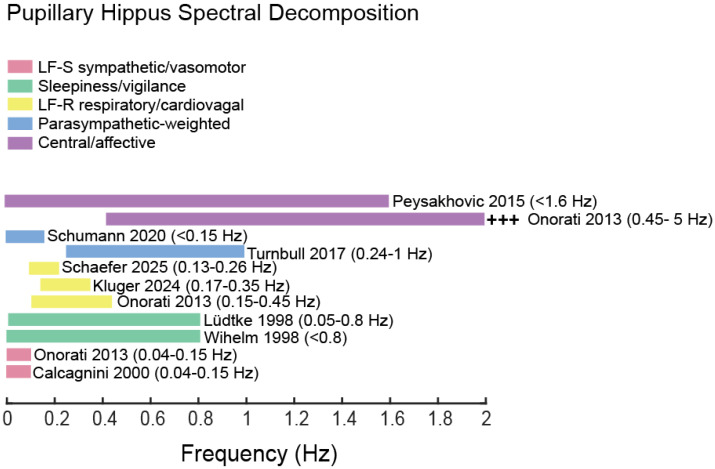
Previously used mappings of hippus frequency bands onto autonomic and higher functions. The diagram shows a summary of various authors who mapped hippus frequency bands onto other physiological concurrent activities. Each horizontal band represents a case of reported frequency interval (in parentheses), color-coded relative to the proposed autonomic or somatic origin in humans. The figure is intended as an illustrative example of the diversity of mappings across studies, not as a prescriptive taxonomy [2,3,35,44,52,54,57,58,59].

Figure 1 summarizes representative studies that assign specific physiological contributions to distinct frequency bands, highlighting the heterogeneity of these attributions. In the following subsections, we examine how different authors have allocated physiological meaning across the spectrum, and in Section 4.7 we synthesize these observations into a paradigm-aware classification.

For physiological context (not depicted in the figure), the canonical HRV bands (LF 0.04–0.15 Hz and HF 0.15–0.40/0.45 Hz) are useful comparative anchors when interpreting pupillary spectra given possible shared autonomic generators; however, they should not be taken as strict equivalences between HRV and pupillary dynamics [48,55,56].

### 4.2. Vigilance and Sleepiness-Related Slow Fluctuations

Under dark steady conditions, pupillary unrest provides a robust biomarker of vigilance decline [46,52,57,60,61]. The PUI summarizes slow pupil diameter fluctuations over several minutes, designed to capture low-frequency oscillations predominantly below <0.8 Hz [46,52,57]. Standards define recording duration (∼11 min) and sampling (∼25 Hz) [19]. Validation studies show strong intra-individual correlations between the PUI and waking EEG δ, θ, and low-α ($\alpha_{1}$) power during sleep deprivation [47]. In separate cohorts, PUI metrics also correlate with vagal HRV indices, such as RMSSD and baroreflex sensitivity [3]. Collectively, these findings delineate a ‘sleepiness–vigilance’ band (0–0.8 Hz) reflecting slow spontaneous pupil oscillations of mixed autonomic origin. Spectral and correlational work links these fluctuations to vagal HRV indices and sympathetic arousal markers, while the broader pupillometry–LC literature suggests they index reduced central arousal.

### 4.3. Low-Frequency Domain (≈0.04–0.33 Hz): Vasomotor–Respiratory Co-Modulation

At these frequencies, pupillary, respiratory, and cardiovascular dynamics can be co-modulated. Borgdorff described respiratory locking of the pupil (inspiratory mydriasis/expiratory miosis), confirmed in humans by Ohtsuka et al.; subsequent work related slow PD oscillations to vasomotor/baroreflex rhythms [22,23,54,58,62,63]. During 0.1 Hz paced breathing, pupil–RESP/HRV coupling becomes prominent [3], consistent with respiratory sinus arrhythmia defined in HRV standards [48,56]. In a large sample, Schumann et al. showed that slow pupillary instability (e.g., PUI < 0.15 Hz) correlates with vagal markers (e.g., RMSSD) and that 0.1 Hz breathing shifts power into low frequencies [3].

In the pupillary respiratory-phase response (PRP) [35], phase-locked coupling between respiration and pupil size persisted under paced breathing (8 and 16 breaths/min; 0.133 and 0.267 Hz). They focused on phase-resolved pupil–respiration coupling rather than on a full frequency-domain decomposition (pupil power spectra or coherence). More recently, Kluger et al. combined pupillometry and respiratory recordings across multiple magnetoencephalography (MEG) datasets and showed that pupil–respiration coherence is selectively enhanced in a narrow low-frequency band (0.17–0.37 Hz) overlapping with both spontaneous hippus (0.19 Hz) and the respiratory fundamental, with stronger coupling at rest than during a visual task and a coherence peak that tracks paced-breathing frequency [59].

In the low-frequency range, sympathetic contributions to pupil dynamics are expected to covary with electrodermal activity (EDA): emotional arousal typically evokes concurrent pupil dilations and skin-conductance responses [64]. Concordantly, under an orthostatic sympathetic test (70° head-up tilt test), Calcagnini et al. observed a relative shift toward LF power in the pupillary spectrum (LF/HF ↑) with respiratory HF coupling preserved, whereas LF coherence with RR/SBP (RR intervals: beat-to-beat heart period derived from the ECG; SBP: systolic blood pressure) emerged only in a subset—consistent with a mixed baroreflex/vasomotor rather than purely sympathetic LF component [58].

Multimodal work confirms that pupillary features covary with autonomic indices (EDA/HRV/RESP) in affective tasks [54,64]. Converging fMRI/invasive physiology indicates that especially the pupil derivative covaries with LC and salience/attentional networks, consistent with central arousal influences on slow pupil-diameter dynamics [16,30]. Thus, within LF, a sympathetic-enriched component (LF-S) is plausible (albeit non-exclusive). Given a recent systematic review reporting inconclusive evidence for robust breathing-to-pupil shaping across tasks, these mappings should be validated within-study [34].

**Operational split.**

- LF-S (0.04–0.10 Hz): slow vasomotor/baroreflex-enriched rhythms (sympathetic-weighted but mixed); sensitive to posture/vascular tone.
- LF-R (∼0.10–0.24/0.33 Hz): respiratory–cardiovagal coupling with phase-locking; upper bound tracks breathing rate and is accentuated by 0.1 Hz paced breathing. Boundaries should be adjusted to the observed breathing-rate distribution and reported explicitly, including respiratory rate, vigilance, illumination, and confirming attributions with PD–RESP/HRV coherence and, where feasible, pharmacology.

### 4.4. Parasympathetic Weighting in the Upper Hippus Band (0.25–1 Hz)

Pharmacological manipulations suggest that spontaneous hippus in the 0.2–1 Hz range is predominantly driven by parasympathetic input. In resting humans, topical parasympatholysis with tropicamide produces a marked (∼70%) reduction in hippus power under both photopic and scotopic conditions, whereas the sympathomimetic phenylephrine substantially dilates the pupil without suppressing unrest, implicating a dominant central parasympathetic generator under steady light [2]. Spectral decompositions of tonic pupil fluctuations further place much of this “hippus-like” variability in an upper band between ∼0.2 and 1 Hz, which overlaps with arousal-related components [54]; depending on breathing rate, this band can partially overlap with respiratory-driven oscillations.

Concordant perioperative studies show that μ-opioid agonists such as fentanyl and remifentanil cause a dose-dependent depression, and in some cases near-abolition, of pupillary unrest in ambient light, with PUAL often reaching near-zero values at opioid concentrations associated with respiratory depression [40,65,66]. These findings support a central origin of hippus in midbrain pupillomotor circuits (periaqueductal gray–Edinger–Westphal complex) and are consistent with opioid modulation of arousal-related neuromodulatory nuclei, including the LC–NE system, and with a rebalancing of parasympathetic–sympathetic influences on the iris.

### 4.5. High-Frequency (>1 Hz): Central/Affective Content

In affective and cognitive-load paradigms, faster components differentiate conditions: Onorati et al. reported robust differences across 0.45–5 Hz between baseline and anger with autonomic covariates [54], consistent with the nonlinear structure in pupillary dynamics [67]. Components up to ∼2 Hz remain physiologically plausible in task-evoked responses. Peysakhovich et al. used frequency-domain analysis of task-evoked pupil responses in an auditory memory task, defining low- (0–1.6 Hz) and high-frequency (1.6–4 Hz) bands data-driven via effect sizes for load and luminance [53]. Memory load selectively increased low-frequency power, while luminance impacted both bands, and an LF/HF ratio was proposed as a luminance-robust index of mental effort [53].

Extending this line of work, Duchowski et al. showed with the Low/High Index of Pupillary Activity (LHIPA) that the relative balance between low- and high-frequency pupillary oscillations decreases systematically with increasing mental effort across mental arithmetic, *n*-back, and eye-typing tasks, reinforcing the view that fast phasic components in the >1 Hz range carry central cognitive–affective load information while remaining particularly sensitive to luminance, gaze angle, and preprocessing choices [68].

### 4.6. Paradigms to Probe Autonomic Content

State manipulations that selectively engage autonomic circuits help to ascribe physiological meaning to bands.

- Paced breathing (6 cpm ≈ 0.1 Hz): emphasizes respiratory–cardiovagal coupling; analyze pupil–respiration/HRV coherence and phase relations.
- Apnea (breath-hold) and cold-face immersion (diving reflex): breath-hold suppresses the 0.1-Hz respiratory drive, helping to separate LF-S from LF-R; cold-face immersion adds vagal bradycardia and peripheral vasoconstriction, useful to test HF shifts and increased complexity [49].
- Pharmacology: tropicamide vs. phenylephrine to probe HF-P; μ-opioid agonists depress pupillary unrest under ambient light.
- PST/drowsiness (darkness and prolonged baselines): emphasizes low-frequency instability (PUI) with high vigilance sensitivity.
- Affective and cognitive-load paradigms: often recruit high-frequency components (typically > ∼0.45–0.5 Hz) and enhanced nonlinear structure in the pupillary signal, in parallel with autonomic covariates. For brief mental-effort tasks, extended high-frequency bands (e.g., 1.6–4 Hz) and LF/HF-style indices have been used to isolate central cognitive–affective load from luminance-driven variance.

Thus, as a method caveat, given inconsistent evidence for generic breathing-to-pupil entrainment across tasks, band attribution should be validated within each paradigm.

### 4.7. Proposed Paradigm-Aware Taxonomy (Operational Use)

We propose a concise paradigm-aware taxonomy of pupillary oscillation bands that integrates autonomic, respiratory, pharmacological, and vigilance evidence. Band limits are operational and context-dependent: they may shift with illumination, breathing rate, vigilance, and recording choices, yet they offer a practical scaffold to design analyses, report methods, and attribute physiology. Validation should combine state manipulations (paced breathing and apnea), pharmacology, and multimodal autonomic recording (HRV/EDA/RESP). Table 2 summarizes candidate bands, primary autonomic weighting, and paradigms best suited to probe each component.

**Method notes**

Overlap across bands is expected as respiratory influence may extend beyond 0.25 Hz; band edges should be pre-registered to the analysis protocol. Validate mappings via state manipulations (breathing, apnea, and cold-face immersion) and pharmacology. For > 1 Hz, ensure high sampling and artifact handling before inferring physiological content.

## 5. Nonlinear Analysis of Pupil Dynamics

Nonlinear indexes complement spectral power by capturing the irregularity and temporal structure of the hippus under steady illumination. These approaches provide insight into the dynamical organization of the autonomic control loops that regulate the pupil beyond what can be inferred from linear measures. Below, a brief overview of the most widely used complexity estimators is provided.

### 5.1. Overview of Nonlinear Estimators

Different approaches and indexes have been introduced to characterize the nonlinear behavior of a time series.

1. **Fractal dimension.** The fractal dimension (*D*) quantifies how the structure of a signal fills its space as the scale of observation changes, reflecting self-similarity and scaling properties. Introduced by Mandelbrot in the context of fractal geometry [69], it has been widely adopted to quantify irregularity in physiological time series. Lower values of *D* correspond to smoother or more regular fluctuations, whereas higher values indicate greater irregularity and richer temporal dynamics.
2. **Approximate entropy (ApEn) and sample entropy (SampEn).** ApEn was introduced by Pincus [26] to quantify regularity in finite-length physiological signals, while SampEn was later proposed by Richman and Moorman [70] to improve consistency across different data lengths and reduce bias due to self-recurrences. Both indexes estimate the likelihood that patterns of observations that are close remain close when extended by one sample, thus measuring the predictability of the signal. Higher entropy values indicate increased irregularity and complexity.
3. **Hurst exponent.** The Hurst exponent (*H*), first described in hydrology by Hurst [71], quantifies the presence of long-term correlations (long-range dependence) in a time series. Values of H≈0.5, H>0.5, and H<0.5 correspond to uncorrelated (random) fluctuations, persistent correlations (positive feedback), and anti-persistence, respectively. In pupillometry, deviations of *H* from 0.5 suggest structured temporal dependencies in autonomic modulation.
4. **Detrended Fluctuation Analysis.** Detrended Fluctuation Analysis (*DFA*), introduced by Peng et al. [72], provides a robust estimation of scaling exponents in non-stationary time series by quantifying the relationship between window size *n* and fluctuation amplitude F(n) after removing local trends. It allows the assessment of self-similarity and long-range correlations across different time scales. The slope of the logF(n) vs. logn plot, α, corresponds to the scaling exponent, which is related to the Hurst exponent under certain conditions.
5. **Recurrence Quantification Analysis (RQA).** Recurrence Quantification Analysis (*RQA*), first formalized by Zbilut and Webber [73], investigates the recurrence of states in the reconstructed phase space of a signal. RQA indexes, such as recurrence rate (RR), determinism (%DET), entropy, and laminarity (LAM), capture the degree of recurrence, predictability, complexity, and laminar behavior of nonlinear dynamics. In pupillometry, RQA enables detection of structured deterministic components that coexist with stochastic variability.

To ensure that nonlinear estimators capture genuine nonlinear information, statistical testing with surrogate data was introduced by Theiler et al. [74]. This approach involves comparing nonlinear indices obtained from the original signal with those computed from randomized surrogates that preserve linear correlations. Significant differences indicate that the analyzed dynamics cannot be explained by linear processes alone.

### 5.2. Methodological Considerations

Reliability of nonlinear estimators depends on preprocessing and parameterization. Short artifact-prone pupillometric recordings benefit from standardized cleaning procedures, including blink and saccade handling, detrending, and interpolation. For ApEn and SampEn, indications on low-frequency detrending, sampling rate, and embedding dimension have been provided in [51], together with a modified version of ApEn that automatically adapts the tolerance threshold. When physiological attribution is of interest, band-specific entropy can be reported alongside spectral power to discriminate the complexity of rhythms dominated by sympathetic (SNS) or parasympathetic (PNS) modulation.

### 5.3. Applications of Nonlinear Estimators to Pupillometry

Several studies have applied nonlinear measures to spontaneous or stimulus-evoked pupil dynamics, highlighting their relevance for characterizing autonomic function and cognitive states.

1. ApEn and SampEn quantify regularity (higher values indicate greater irregularity/complexity) and have been applied to spontaneous pupillary dynamics, which exhibit non-random structured behavior at rest [26,33].
2. In state manipulations, increased entropy has been observed under conditions implying greater autonomic flexibility. For example, pupillary entropy rises during affective activation [33] and during parasympathetic-dominant states such as the diving reflex [49], consistent with enhanced adaptability of autonomic control loops.
3. Different psychophysiological states were induced in 25 subjects [67]: baseline, anger, joy, and sadness. Several nonlinear indexes were compared, including sample entropy, Correlation Dimension (quantifying recurrences), and the Largest Lyapunov Exponent (indicating, when positive, divergent and chaotic dynamics). A piecewise linear regression model was then applied to DFA. The results suggest that (a) pupil size under constant light is characterized by nonlinear dynamics, (b) three distinct long-memory processes exist at different time scales, and (c) autonomic stimulation is partially reflected in nonlinear dynamics.
4. Pupil dynamics were compared during habitual dental occlusion (HDO), considered a mild stimulation of the Autonomous Nervous System (ANS), versus rest position (RP) of the jaw [75]. Linear and nonlinear RQA-based indexes were estimated and used to distinguish between the two conditions. RQA indexes provided additional information on pupil dynamics beyond linear descriptors, enabling discrimination of even subtle autonomic effects.
5. The importance of RQA indexes extracted from the pupillogram (especially %DET) was also found in characterizing patients with obstructive sleep apnea syndrome (OSAS) and temporomandibular disorder [76,77].
6. Decomposition by Empirical Mode Decomposition (EMD) was used to separate LF/HF components of the pupillogram; then, Cross-Recurrence Quantification Analysis (CRQA) was applied to assess the similarity between parasympathetic and sympathetic dynamics by comparing their LF–HF interaction in phase space [78]. Entropy and determinism of these interactions were found to relate to autonomic tone and awareness, confirming CRQA as a tool for studying coupled dynamics.
7. Slow pupil diameter oscillations (f<0.15 Hz), quantified by PUI, were found to reflect parasympathetic modulation [3]. Sympathetic arousal, as detected by skin conductance fluctuations, was associated with nonlinear pupillary dynamics explored by SampEn.
8. Nonlinear methods have also been applied to pupil diameter recorded during stimulation. A robust estimator of the Hurst exponent (*H*) was obtained using non-decimated wavelets and applied to pupil response behavior (PRB) to classify visual deficits [79]. The proposed predictor improved accuracy compared to standard wavelet methods. Moreover, SampEn was applied to the pupillary light reflex (PRL) in glaucoma versus controls (three light intensities) [80], revealing differences in signal complexity between groups and suggesting clinical utility.
9. The fractal dimension of pupil diameter was used as an index of complexity during multitasking [81,82]. It was found to be sensitive to cognitive load and to correlate more strongly with task performance (NASA-TLX) than traditional metrics.
10. The hippus was studied at rest using SampEn and Transfer Entropy (TranEn) to assess complexity and symmetry, respectively [83]. Evidence from surrogate testing supported a nonlinear deterministic process, with inverted U-shaped relationships between complexity/symmetry and baseline. Large pupil diameters and low temporal complexity (SampEn) and symmetry (TranEn) were also associated with Attention-Deficit/Hyperactivity Disorder (ADHD) [84].
11. Clinically, entropy-based pupillometry has shown preliminary discrimination in glaucoma cohorts, Alzheimer’s disease, and ADHD case-control studies, albeit based on small samples and heterogeneous protocols [80,84,85].

## 6. Clinical and Translational Applications

### 6.1. Overview of Clinical Pupillometry

Pupillometry has evolved from a neurophysiological probe to a translational biomarker, offering an objective non-invasive window to central autonomic and noradrenergic function across laboratory and bedside settings [12,86]. In sleep medicine, PST quantifies drowsiness via the PUI, capturing slow oscillations (<∼0.8 Hz) that grow as vigilance declines [46,47,57,60,61]. These measures support evaluation in sleep disorders (e.g., narcolepsy and excessive daytime sleepiness) and in occupational safety contexts requiring objective vigilance assessment [47].

Pupillary dynamics also serve as real-time pharmacological monitors: μ-opioid agonists and anesthetic agents reduce parasympathetic drive and suppress spontaneous unrest, enabling automated tracking of opioid exposure and depth-of-anesthesia [40,65,66]. In parallel, fluctuations in pupil diameter index activity in locus coeruleus–noradrenergic (LC–NE) circuits that govern arousal, attention, and control, with the pupil (and especially its temporal derivative) tracking rapid adrenergic/cholinergic dynamics [11,16,18]. This coupling motivates applications in psychiatry, stress, and cognitive fatigue.

In visual display unit (VDU) fatigue and occupational ergonomics, spectral and model-based analyses of pupil variability detect mental workload and visual fatigue, particularly with high sampling and luminance-independent paradigms [53]. In neurodegeneration, both pupillary light reflex (PLR) features and spontaneous dynamics show alterations in Parkinson’s disease and Alzheimer’s disease (AD), supporting parasympathetic and noradrenergic involvement and suggesting early risk stratification roles [85,87,88,89].

Pupillometry has been applied to concussion and mild traumatic brain injury (mTBI), where chronic cohorts show smaller baseline pupils and delayed, slowed, and reduced PLR responses relative to controls [90,91]. In acute and sports-related brain injury, abnormal Neurological Pupil Index (NPi) values and quantitative pupillometry predict outcome and return-to-play, supporting their use as emerging adjunctive biomarkers across the TBI spectrum [92,93,94].

Additionally, recent neurology literature describes the ”pupillary (hippus) nystagmus” as a clinical sign supporting vestibular migraine diagnosis [95].

Together, these strands illustrate the translational potential of pupillometry as a multifunctional biomarker—linking physiology, pharmacology, and neuropathology—while underscoring the need for standardized acquisition and harmonized spectral/nonlinear analysis to enable robust cross-study comparability.

### 6.2. Glaucoma as a Translational Model

Among neurodegenerative ocular diseases, glaucoma offers a compelling setting in which pupillometric biomarkers may capture both retinal and central autonomic signatures. Beyond classical optic neuropathy, glaucoma entails retinal ganglion cell (RGC) degeneration that can involve non-image-forming (NIF) pathways—particularly ipRGCs projecting to circadian and arousal centers (e.g., suprachiasmatic nucleus (SCN) and LC). Chromatic pupillometry quantifies the afferent melanopsin pathway via the ipRGC-mediated post-illumination pupil response (PIPR), which is reduced in glaucoma and scales with disease severity [96,97,98,99,100,101,102]. By contrast, spontaneous hippus under steady light primarily reflects central autonomic dynamics and should not be conflated with PIPR (see Section 3 for methodological standards). Accordingly, pupillometry can probe both afferent integrity (PLR/ipRGC-mediated PIPR) and centrally modulated spontaneous dynamics. Converging evidence places the LC–NE system at the crossroads of arousal regulation, autonomic control, and neurodegeneration: tau aggregation and noradrenergic dysfunction in the LC emerge early in AD, preceding cortical pathology by decades [87,88]. Given this shared susceptibility, pupillary changes in glaucoma and AD may partly reflect common noradrenergic and neuroinflammatory mechanisms [103,104].

Clinically, glaucoma cohorts show asymmetric PLR, relative afferent pupillary defects that correlate with inter-eye asymmetry of RNFL thickness and visual field loss, reduced constriction amplitude/velocity, and latency delays [96,97,98,99,100,101,102]. The fractal dimension and sample entropy of pupillary signals of glaucoma patients are higher than the age-matched and young control groups [80], while IpRGC-mediated post-illumination pupil response (PIPR) is attenuated in glaucoma and scales with structural and functional disease severity [96,101,105]. Resting-state fMRI further reports altered LC activity in primary open-angle glaucoma (POAG), with low-frequency metrics correlating with cup-to-disc ratio, RNFL thickness, and visual function [106]. Regarding sleep and circadian phenotypes, multiple cohorts link reduced PIPR to poorer sleep quality in glaucoma—most robustly in severe disease (LIGHT Study) [105,107]. In a cross-sectional study combining chromatic pupillometry with overnight polysomnography, Gracitelli et al. showed that glaucoma patients with reduced blue-light PIPR had shorter total sleep time, lower sleep efficiency, more nocturnal arousal and oxygen desaturation, and that these abnormalities correlated with both ipRGC-mediated pupillary responses and RNFL thinning [108]. However, a small normal-tension glaucoma (NTG) cohort found no PSQI difference versus controls despite reduced PIPR, underscoring heterogeneity and limited power in the early/milder stages [109]. Therefore, evidence supports associations between ipRGC dysfunction and sleep disturbance, but causality is unproven.

Altogether, these data position pupillometry as a candidate biomarker indexing both retinal (ipRGC) and central autonomic (LC–NE) alterations in glaucoma. Chromatic pupillometry (PIPR) provides an afferent index of ipRGC dysfunction, whereas hippus-based spectral/complexity metrics may capture central autonomic alterations—two complementary but mechanistically distinct readouts. However, despite promising group-level differences, the current discriminative performance (AUCs) from computerized pupillometry is moderate and insufficient for stand-alone diagnosis or triage; standardized acquisition (fs, filters, anti-aliasing, blink handling, and PFE correction), harmonized spectral/complexity analysis, and larger multicenter cohorts are required. Prospective and interventional evidence is emerging; e.g., a pilot of increased daytime bright-light exposure in glaucoma reported greater PIPR and improved subjective sleep, but replication in adequately powered RCTs is needed [110]. Advancing clinical utility will require standardized spectral and complexity metrics, larger multicenter cohorts, and integration with imaging biomarkers (e.g., OCT and LC-sensitive MRI) to validate pupillary oscillations as early indicators and monitoring tools in glaucomatous (and broader neurodegenerative) progression.

## 7. Challenges and Future Directions

The key barriers include heterogeneous band definitions; confounds from luminance, respiration, and vigilance; drug and temperature effects; and limited cross-device comparability. Overall, complexity measures appear promising as adjunct biomarkers, best used together with spectral/physiological features.

We recommend joint reporting of spectral (LF/HF) and complexity (ApEn/SampEn) metrics and, where feasible, controlled physiological perturbations to tease apart autonomic components. Combining these metrics with system identification and machine learning may improve effect sizes and diagnostic AUCs in selected indications.

## 8. Conclusions

Hippus is a multiscale signal reflecting sympathetic/parasympathetic interplay and LC-mediated arousal. Spectral and complexity analyses, particularly under strong autonomic probes, offer complementary views on this physiology. With standardized protocols and cautious interpretation, pupillometry can mature from a research tool into a clinically meaningful biomarker.

## Figures and Tables

**Table 2:** Summary of frequency bands associated with pupillary oscillations (hippus) and their physiological correlates.

Band Label	Frequency Range (Hz)	Dominant Modulation (Context-Dependent)	Typical Paradigms/Checks
VIG (Vigilance/Drowsiness)	<∼0.8	Mixed autonomic instability during low arousal	Dark room; prolonged baselines; time-of-day and alertness reporting
LF-S (Sympathetic- weighted, slow)	0.04–0.10	Baroreflex and vasomotor rhythms	Baseline; apnea to separate from LF-R; EDA concurrent recording; posture/vascular tone control
LF-R (Respiratory/Cardiovagal)	∼0.10–0.24 (≤0.33)	Respiratory sinus arrhythmia coherence	Paced breathing (0.1 Hz, 6 cpm); PD–RESP/HRV coherence; adapt to breathing rate
HF-P (Parasympathetic-Weighted)	0.25–1.0	Sensitive to parasympatholysis (tropicamide)	Pharmacology (tropicamide vs. phenylephrine); steady illumination; report respiratory rate
HF-X (Central/Affective)	1.0–2.0 (extendable to ∼4 Hz in cognitive paradigms)	Central/affective content; ↑ nonlinearity and entropy	Affective/cognitive tasks

## Data Availability

No new data were created or analyzed in this study.

## Abbreviations

The following abbreviations are used in this manuscript:

AD	Alzheimer’s Disease
ADHD	Attention-Deficit/Hyperactivity Disorder
ANS	Autonomic Nervous System
ApEn	Approximate Entropy
BF	Basal Forebrain
cd·m^−2^	Candelas Per Square Meter (luminance)
cpm	Cycles Per Minute (breathing rate; 6 cpm ≈ 0.1 Hz)
CRQA	Cross-Recurrence Quantification Analysis
DFA	Detrended Fluctuation Analysis
ECG	Electrocardiogram
EDA	Electrodermal Activity
EEG	Electroencephalography
EMD	Empirical Mode Decomposition
fMRI	Functional Magnetic Resonance Imaging
HF	High Frequency (spectral band)
HF-P	High-Frequency Parasympathetic-Weighted Band (∼0.25–1 Hz)
HF-X	High-Frequency Fast Central/Affective Band (∼1–2 Hz; up to ∼4 Hz in tasks)
HRV	Heart Rate Variability
IR	Infrared (illuminator wavelength)
ipRGC(s)	Intrinsically Photosensitive Retinal Ganglion Cell(s)
LC	Locus Coeruleus
LC–NE	Locus Coeruleus–Noradrenergic System
LF	Low Frequency (Spectral Band)
LF-R	Low-Frequency Respiratory/Cardiovagal Band (∼0.10–0.24/0.33 Hz)
LF-S	Low-Frequency Sympathetic-Weighted/Vasomotor Band (∼0.04–0.10 Hz)
ML	Machine Learning
MRI	Magnetic Resonance Imaging
NASA-TLX	NASA Task Load Index
NIF	Non-Image-Forming (visual pathways)
NPi	Neurological Pupil Index
NTG	Normal-Tension Glaucoma
OCT	Optical Coherence Tomography
OSAS	Obstructive Sleep Apnea Syndrome
PD	Pupil Diameter
PFE	Pupil-Foreshortening Error
PIPR	Post-Illumination Pupil Response
PLR	Pupillary Light Reflex
POAG	Primary Open-Angle Glaucoma
PNS	Parasympathetic Nervous System
PPG	Photoplethysmography
PSD	Power Spectral Density
PST	Pupillographic Sleepiness Testing
PSQI	Pittsburgh Sleep Quality Index
PUI	Pupillary Unrest Index
PWM	Pulse-Width Modulation (of light sources/displays)
RCT	Randomized Controlled Trial
RESP	Respiration Signal (belt/flow)
RGC	Retinal Ganglion Cell
RMSSD	Root Mean Square of Successive Differences
RNFL	Retinal Nerve Fiber Layer
RQA	Recurrence Quantification Analysis
SampEn	Sample Entropy
SNS	Sympathetic Nervous System
TLM	Temporal Light Modulation
TranEn	Transfer Entropy
VDU	Visual Display Unit (fatigue paradigm)
VIG	Vigilance/Drowsiness Band (<∼0.8 Hz)
mTBI	Mild Traumatic Brain Injury
